# Hypothiocyanite and Hypothiocyanite/Lactoferrin Mixture Exhibit Virucidal Activity In Vitro against SARS-CoV-2

**DOI:** 10.3390/pathogens10020233

**Published:** 2021-02-19

**Authors:** Luca Cegolon, Mattia Mirandola, Claudio Salaris, Maria Vittoria Salvati, Giuseppe Mastrangelo, Cristiano Salata

**Affiliations:** 1 Public Health Department, Local Health Unit N.2 “Marca Trevigiana”, 31100 Treviso, Italy; 2 Department of Molecular Medicine, University of Padova, 35121 Padova, Italy; mattia.mirandola@gmail.com (M.M.); claudio.salaris@studenti.unipd.it (C.S.); mariavittoria.salvati@phd.unipd.it (M.V.S.); 3 Department of Cardiac, Thoracic, Vascular Sciences & Public Health, University of Padova, 35121 Padova, Italy; giuseppe.mastrangelo@unipd.it

**Keywords:** hypothiocyanite, lactoferrin, lactoperoxidase system, ALX-009, SARS-CoV-2, COVID-19

## Abstract

SARS-CoV-2 replicates efficiently in the upper airways during the prodromal stage, resulting in environmental viral shedding from patients with active COVID-19 as well as from asymptomatic individuals. There is a need to find pharmacological interventions to mitigate the spread of COVID-19. Hypothiocyanite and lactoferrin are molecules of the innate immune system with a large spectrum cidal activity. The Food and Drug Administration and the European Medicines Agency designated the hypothiocyanite and lactoferrin combination as an orphan drug. We report an in vitro study showing that micromolar concentrations of hypothiocyanite exhibit dose- and time-dependent virucidal activity against SARS-CoV-2 and that the latter is slightly enhanced by the simultaneous presence of lactoferrin.

## 1. Introduction

SARS-CoV-2 enters target cells binding the angiotensin-converting enzyme 2 (ACE2) receptor, widely expressed in human airways, particularly by epithelial cells of the nasal cavity and type II pneumocytes of the lung alveoli [1,2]. COVID-19 physiopathology can be classified into three clinical stages, depending on the main site of the infection [3]. At the initial asymptomatic stages, SARS-CoV-2 infection is predominantly located in the nasal cavity, where it elicits a contained local innate immune response. In the second mild symptomatic stage, COVID-19 mainly affects the pseudostratified epithelium of the upper respiratory tract, which can recover because the basal cells are spared. However, COVID-19 may cause a more severe reaction in the smaller branches of the bronchial airways, where club cells are likely infected. In the third life-threatening stage, SARS-CoV-2 spreads into the pulmonary alveoli by infecting type II pneumocytes expressing the ACE2 receptor.

Whilst treatment options for critically ill COVID-19 patients requiring hospitalization are now available, with corticosteroids emerging as the most effective one [4], there is a noteworthy lack of effective remedies against mild to moderate disease. It is believed that treatments with few adverse effects and that are easy to administer in outpatient settings, combined with an effective vaccine, could end the ongoing COVID-19 pandemic [5]. Molecules derived from the innate immune system, such as hypothiocyanite (OSCN^−^; structural formula ^−^O-S-C≡N) and lactoferrin (LF), represent promising candidates due to their broad-spectrum antimicrobial and antiviral activity [6,7].

Interestingly, Alaxia SAS has recently developed a solution of the above two molecules combined (ALX-009) for the treatment of bacterial infections in cystic fibrosis patients [8]. The solution was easily administered by inhalation of aerosol in a phase I clinical trial (NCT02598999 of 2018) to healthy volunteers and patients affected by cystic fibrosis. The combination of OSCN^−^ and LF had already been designated as an orphan drug for treatment of cystic fibrosis by the US Food and Drug Administration and the European Medicines Agency in May 2009 [9]. It is conceivable that aerosol topical administration of OSCN^−^ and LF combined might effectively inactivate free SARS-CoV-2 virions nearing the epithelium from outside or the ones being released from infected cells, thus mitigating or preventing the spread of the infection in the host tissues as well as its propagation to further susceptible individuals.

In view of the above, we conducted a study with the aim of testing the in vitro virucidal activity of OSCN^−^ and of a combination of OSCN^−^ and LF.

## 2. Results

### Virucidal Activity of OSCN^−^ and OSCN^−^/LF

**Figure 1:**
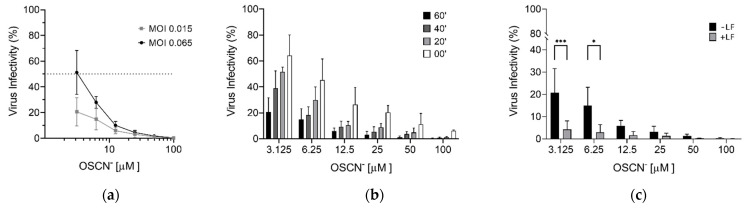
OSCN^−^ and OSCN^−^/LF virucidal activity against the pseudovirus VSV-S diluted in Minimum Essential Medium (MEM). Infection of Vero cells was evaluated measuring the activity of the VSV-S encoded luciferase. (**a**) Efficiency of pseudovirus infection at MOI 0.065 and 0.015 FFU/mL after preincubation with different OSCN^−^ concentrations for 1 h at 37 °C; (**b**) evaluation of the virucidal activity of OSCN^−^ after pseudovirus treatment for 0, 20, 40, and 60 min at 37 °C before the infection of target cells at MOI 0.015; (**c**) comparison between OSCN^−^ and OSCN^−^ + LF virucidal activity after 1 h of preincubation of VSV-S and before cell infection at MOI 0.015. Data (mean ± SD, N = 3, experiments in duplicate) are percentages of no drug, set as 100% (* = *p* < 0.05; *** = *p* < 0.001).

The virucidal activity of OSCN^−^ was firstly investigated using a recombinant vesicular stomatitis virus (rVSV), encoding the reported gene luciferase instead of the viral glycoprotein. The rVSV can be easily manipulated under biosafety level 2 conditions and pseudotyped by the S protein (rVSV-S). The tropism of the rVSV-S is dictated by the heterologous S envelope, and it represents an excellent surrogate of SARS-CoV-2 to study the virus entry and the viral neutralization [10,11,12]. The rVSV-S was therefore incubated with different OSCN^−^ concentrations for 60 min at 37 °C (preincubation), and then Vero cells were infected at multiplicity of infection (MOI) 0.065 and 0.015 FFU/cell. Sixteen hours post-infection, cells were lysed and the luciferase expression was evaluated as a measure of viral infection. As shown in Figure 1a, viral infection was inhibited in a dose-dependent manner with 4.64 μM as the concentration capable of reducing the viral infectivity by 50% (IC_50_) at the higher MOI. Moreover, more than 80% of rVSV-S infection was inhibited at the lower MOI. When performing the infection at the MOI 0.015 FFU/mL, it became evident that the efficacy of the virucidal activity of OSCN^−^ was also time-dependent in all conditions tested (preincubation: 60, 40, and 20 min; without preincubation (0 min) adding OSCN^−^ during the infection of target cells) (Figure 1b). In particular, when the rVSV-S was incubated with OSCN^−^ before the infection, we observed a reduction of the viral infectivity of more than 50% up to the concentration of 6.25 μM (Figure 1b). Without preincubation (0 min), a reduction of virus infectivity higher than 50% was observed starting from OSCN^−^ concentration of 12.5 μM.

**Figure 2:**
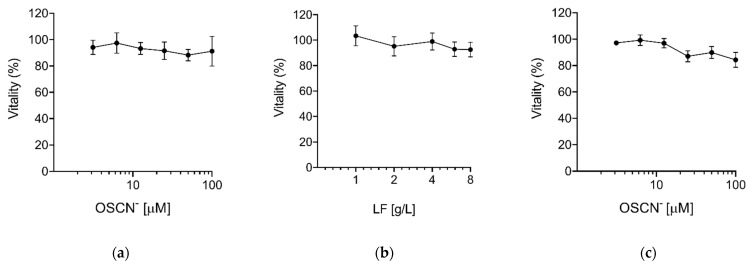
Cytotoxicity of OSCN^−^ and LF. The cytotoxicity of (**a**) OSCN^−^, (**b**) LF, and (**c**) OSCN^−^+ LF (4 g/L) diluted in MEM was evaluated on Vero cells after 24 h of treatment using the MTT assay. Data (mean ± SD, N = 3, experiments in quadruplicate) are percentages of no drug, set as 100%.

We also investigated the virucidal activity of OSCN^−^ and LF combined. Preliminary experiments with LF showed that concentrations higher than 1 g/L were required to inhibit the viral infection (data not shown). Therefore, we selected a concentration of 4 g/L that was close to that previously used with OSCN^−^ in ALX-009 against bacteria [8]. The combination OSCN^−^/LF significantly increased the virucidal activity despite using lower doses of OSCN^−^, achieving an inhibition of viral infection > 90% in comparison to 80–85% with OSCN^−^ alone (Figure 1c) or 25% with LF alone (data not shown). These data suggest that, in our experimental conditions, OSCN^−^ is the main virucidal agent, whereas at lower concentrations of OSCN^−^ the presence of LF improves virus inactivation. As the addition of OSCN^−^ and LF was not toxic to Vero cells, as shown by the outcome of the test for metabolic activity (MTT) conducted 24 h after their exposure to both reagents, we concluded that the inhibition of the viral infectivity was due to the ability of both compounds to interfere with the capacity of the virus to infect cells (Figure 2).

**Figure 3:**
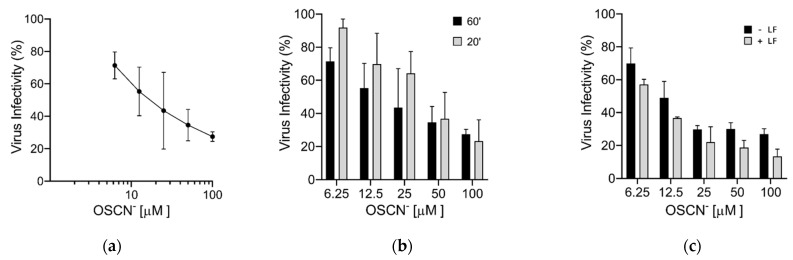
Virucidal activity of OSCN^−^ and OSCN^−^/LF against SARS-CoV-2. SARS-CoV-2 was diluted in MEM and incubated for 1 h at 37 °C with only OSCN^−^ or supplemented with LF before infection of Vero-E6 cells. The reduction of infectivity was evaluated by plaque assay. (**a**) Virucidal effect of OSCN^−^; (**b**) comparison between different times of virus-OSCN^−^ exposure on the efficiency of the virucidal activity; (**c**) evaluation of the combination OSCN^−^+ LF on the virucidal activity. Data (mean ± SD, N = 3, experiments in duplicate) are percentages of no drug, set as 100%.

Finally, we verified the virucidal activity of OSCN^−^ and OSCN^−^/LF displayed against the VSV-S by using the real SARS-CoV-2. After virus-compound incubation for 60 min, 10-fold dilutions of virus-compound mix were inoculated onto Vero-E6 cells (used for virus isolation and propagation) and the reduction of plaque generation was evaluated. Results confirmed the dose-dependent virucidal activity of OSCN^−^ (IC_50_ ~11 μM) and demonstrated that higher doses of OSCN^−^ were required to inhibit SARS-CoV-2 infection with an incubation of 20 min (Figure 3a,b). In contrast to rVSV-S, the LF enhancement of the virucidal activity of OSCN^−^ was significantly less pronounced for SARS-CoV-2 (Figure 3c).

Overall, our results indicate that OSCN^−^ has a virucidal activity against SARS-CoV-2, and that the combination of OSCN^−^/LF slightly improved the virucidal effect of OSCN^−^.

## 3. Discussion

We evaluated the virucidal activity of OSCN^−^ and OSCN^−^/LF mixture against SARS-CoV-2. In our experimental conditions, OSCN^−^ had evident in vitro virucidal effect against SARS-CoV-2.

Three components are mixed in the airway lumen to produce the biocidal compound OSCN^−^: lactoperoxidase (LPO), secreted by the serous cells of the submucosal glands and by goblet cells; the thiocyanate anion (SCN^−^), delivered by the duct cells of submucosal gland; and hydrogen peroxide (H_2_O_2_), released by epithelial cells [13]. Unlike in the trachea, in the main (third- and fifth-generation) bronchi, LPO mRNA was nearly absent in the lung parenchyma, suggesting lack of OSCN^−^ production in the alveoli [14]. Furthermore, due to the need to enhance gas exchange, epithelial alveolar cells cannot contain strong protective structures and hence are weak, fragile, and more vulnerable to viral attacks. Since the biocidal effect of OSCN^−^ takes place in the airway lumen, the aerosol administration of OSCN^−^ may increase the inactivation of viral particles in compartments of the respiratory tract lacking endogenous OSCN^−^ synthesis. Of course, only an in vivo study can validate our hypothesis by evaluating the antiviral efficacy in appropriate animal models.

Despite strong evidence of SARS-CoV-2 inactivation, the exact virucidal mechanism of OSCN^−^ is still unknown. Ozone at high doses has been shown to inactivate SARS-CoV-2 by oxidative stress through ozone decomposition products and oxidation of double bonds of viral lipids, proteins, and amino acids, leading to the formation of reactive radicals (RCOO−) [15,16]. Indirect modes of action further propagate the oxidation through a chain reaction [17]. Enveloped viruses—such as vesicular stomatitis Indiana virus, vaccinia virus, influenza A virus, and certain strains of type 1 herpes simplex virus—are more sensitive to ozone, whereas non-enveloped adenovirus type 2 was more resistant to this gas [18,19]. It is therefore plausible that irreversible oxidative damage of the lipid components of the viral envelope or of the nucleoproteins could also be the mechanism of virucidal activity against SARS-CoV-2 of OSCN^−^, which is a less potent (but also less damaging) oxidizing agent than ozone. As already shown, LPO yields OSCN^−^ in the presence of SCN^−^; OSCN^−^ in turn reacts with the thiol moiety of peptides or proteins (R-SH) generating sulfenyl thiocyanate (R-S-SCN), which, upon addition of one molecule of H_2_O, produces sulfenic acid (R-S-OH) as well as regenerating SCN^−^ at the end of the cycle [6]. The LPO cycle thus extends the duration of effects of OSCN^−^ beyond the limited half-life (about 1 h) of this compound, increasing the efficacy of its antiviral activity over time [20]. Sulphydryl oxidation determines inhibition of numerous enzymes containing the amino acid cysteine, abundant in coronavirus spike proteins, which are an excellent target for the inactivating activity of OSCN^−^ [17]. This mechanism of action could partially explain the difference of IC_50_ values obtained with the pseudovirus and SARS-CoV-2. Since the amount of rVSV-S particles used in our experimental setting was lower (4 × 10^4^ FFU) than the amount of SARS-CoV-2 particles (1 × 10^5^ PFU), the inactivating effect of OSCN^−^ on rVSV-S was stronger as compare to SARS-CoV-2.

The OSCN^−^ concentration used in our experimental conditions was up to 100 µM, higher than that reported in human saliva (20–60 µM) or in resting (31 µM), stimulated whole saliva (25 µM), and parotid saliva (30 µM) [21,22]. Our data suggested that physiological OSCN^−^ concentrations could inhibit at least 50% of SARS-CoV-2 infection. However, OSCN^−^ and bovine LF concentrations in ALX-009 were remarkably higher than those used in our experimental setting, reaching values of 3500 µM and 8 g/L, respectively, which therefore should likely maximize their respective virucidal activity [8].

We have previously demonstrated that OSCN^−^ inhibits A(H1N1)pdm09 influenza virus infection, as a similar IC_50_ than that observed for rVSV-S was obtained when the virus was challenged with OSCN^−^ for 60 min at 37 °C before infection [23]. Thereafter, the antiviral activity of OSCN^−^ was also tested against several other types of influenza virus, confirming a strain-independent virucidal effect [24,25]. The higher IC_50_ of SARS-CoV-2 in comparison to influenza viruses does not necessarily imply a lower susceptibility to OSCN^−^, but may depend on different experimental settings. Thus, the IC_50_ values reported by studies that have used different viral loads, OSCN^−^/virus concentrations, and media might not be directly comparable. However, the overall results indicate a potent and potentially wide range of antiviral activity of OSCN^−^ against respiratory viruses.

LF, one of the most abundant antimicrobial proteins occurring in normal airway secretions [26,27], seems to improve the inhibition of the viral infection only at lower OSCN^−^ concentrations. The antiviral mechanism of LF is based on the ability to prevent the virus from anchoring targeted cells [28]. In particular, LF binds with heparan sulfate proteoglycans (HSPGs), which are cell-surface and extracellular matrix macromolecules acting as an attachment factor for many viruses including SARS-CoV-1. LF blocks the infection of SARS-CoV-1 by competing with the virus for HSPGs, therefore preventing the virus from binding to the surface of target cells [28]. Moreover, it has been shown that LF can partially inhibit SARS-CoV-2 infection in Caco-2 cells preincubated for three hours before the infection [29]. However, in our experimental conditions, it was difficult to detect the antiviral activity of LF, as this requires a longer time of exposure to target cells. Different experimental conditions could allow a better evaluation of the LF effect on SARS-CoV-2 infection, with and without the simultaneous presence of OSCN^−^. We can, though, speculate that LF may contribute to the antiviral activity: while OSCN^−^ inactivates the viral particles, LF prevents the virus from binding to target cells.

In conclusion, our results indicate that OSCN^−^ has a virucidal activity against SARS-CoV-2, and LF might increase its antiviral efficacy. Although the in vitro findings require an in vivo validation, the LF and OSCN^−^ combination may have a relevant clinical impact by reducing the diffusion of infection in the host tissues as well as the spread of SARS-CoV-2 particles to new susceptible hosts. Given its proven broad-spectrum virucidal activity, OSCN^−^ may turn out to be beneficial also against the emerging variants of SARS-CoV-2.

## 4. Materials and Methods

### 4.1. Cell Culture and Viruses

Vero (African green monkey kidney cells, ATCC^®^ CCL-81), Vero E6 (ATCC^®^ CRL1586), and HEK293T (ATCC^®^ CRL-11268TM) cells were grown in Dulbecco’s modified Eagle’s medium (D-MEM) containing 10% heat-inactivated fetal bovine serum (FBSi). Cells were maintained in a 5% CO_2_ incubator at 37° C, routinely checked for mycoplasma and confirmed negative. Culture medium and FBSi were obtained from Gibco (Thermofisher, Italy).

SARS-CoV-2 (Genbank: MW000351) and the rVSVΔG-Luc, a recombinant vesicular stomatitis virus containing the gene encoding for the luciferase protein in place of the VSV-G gene [12], were used in the experiments.

### 4.2. Virus Stock Preparation and Titration

Vero E6 cells were seeded in T175 flasks and then infected with SARS-CoV-2 at the multiplicity of infection (MOI) of ~0.1. At 72–96 h post-infection (p.i.), supernatants were collected, centrifuged at 2300 rpm for 10 min, then stored in aliquots at −80 °C. Viral titer was determined by plaque assay on Vero E6 cells seeded on 24-well plates. Tenfold virus dilutions were prepared in DMEM and inoculated on confluent Vero-E6 cells for 1 h at 37 °C. Thereafter, virus inoculum was removed from each well and cells were overlaid with 300 µL of 0.6% carboxymethylcellulose (Merck, Italy) diluted in DMEM supplemented with 2% FBSi. Seventy-two hours p.i., cells were fixed adding 300 µL of 5% formaldehyde (Merck, Italy) in PBS 1× for 30 min at room temperature. Then, cells were stained with crystal violet in 20% ethanol. Virus titer was measured as plaque-forming units per milliliter (PFU/mL) based on the plaques formed in cell culture upon infection. All studies with viable SARS-CoV-2 were performed in the certified BSL3 laboratory.

For SARS-CoV-2-pseudotyped VSV production (rVSV-S), HEK293T cells were seeded in T175 flasks and then transfected by calcium phosphate-DNA precipitation with 40 µg of pSARS-CoV-2-spike plasmid. After 24 h, cells were infected with the rVSVΔG-Luc virus at MOI 4 fluorescent focus-forming units (FFU)/cell. Sixteen hours p.i., cell culture supernatants were harvested and cell debris were cleared by centrifugation (2300 rpm for 7 min at 4 °C). Thereafter, virus particles were pelleted by ultracentrifugation on a 20% p/v a sucrose cushion (27,000 rpm for 150 min at 4 °C) in a Beckmann (Beckman Coulter Italia, Italy) SW 28 Ti swinging-bucket rotor. Pellets were resuspended in 1 mL of ice-cold PBS1X/tube and mixed. Subsequently, the virus was aliquoted and stored at −80 °C until use.

Titration of virus was determined by immunofluorescence on Vero cells seeded on 96-well plates. Viral stock was 10-fold serially diluted in DMEM and inoculated on confluent Vero cells for 1 h at 37 °C. Cells were then washed and DMEM supplemented with 10% FBSi was added. After 18 h, cells were fixed with precooled methanol-acetone for 1 h at −20 °C. Immune staining was performed by incubation with 1:3000 anti-VSV-M monoclonal antibody (Kerafast, Boston, MA, USA) on the infected cells for 90 min at 37 °C, followed by incubation with 1:1000 anti-rabbit Alexa Fluor^®^ 488 (Thermo Fisher Scientific, Italy) for 60 min at 37 °C. The fluorescent foci in each well were counted and viral titer was expressed as FFU/mL [30].

### 4.3. Compounds Preparation and Cytotoxicity Assay

**Figure 4:**
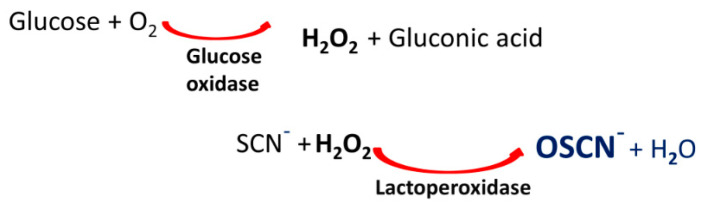
OSCN^−^ extemporaneous production via a two-step biocatalytic pathway.

OSCN^−^ solution was prepared via enzymatic reaction with an automated equipment EOLEASE^®^ (manufactured by Alaxia SAS, Lyon, France), obtaining an enzyme-free OSCN^−^ solution at 3500 ± 10% µM in phosphate buffer (Na_2_HPO_4_/KH_2_PO_4_) at pH 7.5 ± 0.1. The OSCN^−^ was extemporaneously produced via a two-step biocatalytic pathway (Figure 4). The enzyme-free OSCN^−^ solution was then obtained by removing the enzymes from the mixture through a single-use dialysis micromodule (ultrafiltration membrane to eliminate enzymes) to obtain the enzyme-free OSCN^−^ solution in a prefilled removable container. Due to its intrinsic reactivity, each solution freshly prepared was used alone or combined with lactoferrin within 15 min after preparation. Alaxia SAS provided reagents for the OSCN^−^ production.

Pharma-grade lyophilized lactoferrin (purity > 98%) was made available by Alaxia SAS, as sterile vials. Lyophilized lactoferrin was reconstituted as solution with 0.9% sodium chloride solution at 10 g/L.

Compound dilutions for virus treatment were performed in Minimum Essential Media (MEM) purchased by Gibco (Thermofisher, Italy).

Cytotoxicity of OSCN^−^ and LF was determined on Vero and Vero-E6 cells after 24 h of treatment. Cell viability was tested with an assay based on the reduction of a tetrazolium salt (MTT Cell Proliferation Assay, Thermofisher, Italy) in a 96-well plate format according to the manufacturer’s instructions.

### 4.4. Virucidal Assay

To evaluate the OSCN^−^ and LF virucidal activity, 4 × 10^4^ FFU of rVSV-S were incubated for 1 h at 37 °C with 0–3.125–6.25–12.5–25–50 and 100 µM of OSCN^−^ (final volume 350 µL) with or without 4 g/L of LF. Positive control (mock sample) was treated with the solution used to prepare the OSCN^−^ simulating the max OSCN^−^ concentration. Next, Vero cells seeded on 96-well plates were infected for 1 h at 37 °C. Eighteen hours later, infection was evaluated measuring the relative light unit (RLU) with a VICTOR Multilabel Plate Reader (PerkinElmer Italia, Italy) using the Steady-Glo^®^ Luciferase Assay System (Promega, Italy).

In the case of SARS-CoV-2, 1 × 10^5^ PFU were incubated for 1 h at 37 °C with 0–6.25–12.5–25–50 and 100 µM of OSCN^−^ (final volume 300 µL) with or without 4 g/L of LF. Next, 10-fold virus dilutions were prepared in MEM and processed as above reported for the plaque assay. Viral titer was calculated for each sample and the virucidal activity was measured evaluating the efficiency of the infection in comparison to the mock treated control.

### 4.5. Statistical Analysis

All the experiments were performed in duplicate in at least three independent biological replicates. Statistical analysis was carried out with GraphPad Prism 8 software package (GraphPad Software, San Diego, CA, USA), employing the one-way ANOVA test. The threshold for statistical significance was *p* < 0.05. All details on sample size and *p* values for each experiment are provided in the relevant figure or its legend. Curve fitting was performed to determine IC_50_ values using a sigmoidal 4PL model in GraphPad Prism 8 software.

## Data Availability

Publicly available datasets were analyzed in this study. This data can be found here: http://researchdata.cab.unipd.it/—search for “Data referring to the paper: Hypothiocyanite and Hypothiocyanite/Lactoferrin Mixture Exhibit Virucidal Activity In Vitro against SARS-CoV-2”.
